# Fluoroindate glasses co-doped with Pr^3+^/Er^3+^ for near-infrared luminescence applications

**DOI:** 10.1038/s41598-020-77943-w

**Published:** 2020-12-03

**Authors:** Wojciech A. Pisarski, Joanna Pisarska, Marta Kuwik, Marcin Kochanowicz, Jacek Żmojda, Piotr Miluski, Agata Baranowska, Jan Dorosz, Magdalena Leśniak, Dominik Dorosz

**Affiliations:** 1 Institute of Chemistry, University of Silesia, Szkolna 9, 40-007 Katowice, Poland; 2 Bialystok University of Technology, Wiejska 45D Street, 15-351 Bialystok, Poland; 3 AGH University of Science and Technology, 30 Mickiewicza Av, 30-059 Krakow, Poland

**Keywords:** Lasers, LEDs and light sources, Materials for optics, Optical materials and structures

## Abstract

Fluoroindate glasses co-doped with Pr^3+^/Er^3+^ ions were synthesized and their near-infrared luminescence properties have been examined under selective excitation wavelengths. For the Pr^3+^/Er^3+^ co-doped glass samples several radiative and nonradiative relaxation channels and their mechanisms are proposed under direct excitation of Pr^3+^ and/or Er^3+^. The energy transfer processes between Pr^3+^ and Er^3+^ ions in fluoroindate glasses were identified. In particular, broadband near-infrared luminescence (FWHM = 278 nm) associated to the ^1^G_4_ → ^3^H_5_ (Pr^3+^), ^1^D_2_ → ^1^G_4_ (Pr^3+^) and ^4^I_13/2_ → ^4^I_15/2_ (Er^3+^) transitions of rare earth ions in fluoroindate glass is successfully observed under direct excitation at 483 nm. Near-infrared luminescence spectra and their decays for glass samples co-doped with Pr^3+^/Er^3+^ are compared to the experimental results obtained for fluoroindate glasses singly doped with rare earth ions.

## Introduction

Fluoroindate glasses belong to the low-phonon ***H***eavy ***M***etal ***F***luoride ***G***lass (*HMFG*) family, which have been extensively studied for their numerous optical applications. From literature data it is well-known that fluoroindate glasses containing rare earth ions with their lower phonon energies close to 510 cm^−1^ are promising materials for up-conversion luminescence applications. For example, the laser based on the orange-to-ultraviolet conversion in a Nd^3+^ doped fluoroindate glass powder has been well demonstrated^1^. These effects are practically not possible to obtain in Nd^3+^ doped oxide or oxyfluoride glass host matrices. Generally, the efficient up-conversion luminescence of Pr^3+^, Tm^3+^ and Ho^3+^ ions in fluoroindate glasses^2–4^ was successfully observed. Moreover, the up-conversion luminescence spectra of Pr^3+^, Ho^3+^ and Tm^3+^ ions in fluoroindate glasses^5–9^ are enhanced drastically in the presence of Yb^3+^. In the later system^9^, i.e. Tm^3+^/Yb^3+^ co-doped fluoroindate glass, the blue up-conversion luminescence of Tm^3+^ ions is highly increased with Yb^3+^ concentration. For the optimum Tm^3+^ concentration (0.5 mol%), the up-conversion emission intensity was increased by a factor about 100 by co-doping with 2.25 mol% of Yb^3+^. In particular, fluoroindate glasses singly doped with Er^3+^ ions and doubly doped with Er^3+^/Yb^3+^ ions have been examined for up-conversion luminescence^10–13^, which was measured under 790 nm, 980 nm or 1480 nm laser excitation. The up-conversion luminescence processes of Er^3+^ were analyzed with activator concentration, temperature, pumping wavelength and power of diode laser used as the excitation source. In general, the up-conversion results in a strong green emission and weaker blue and red emissions and the Yb^3+^ co-doping will certainly increase the efficiency of an up-conversion-based optical device. Further investigations confirmed this hypothesis. An intensity enhancement of 4.5 for the green up-conversion luminescence in Er^3+^/Yb^3+^ co-doped fluoroindate glass has been obtained by focusing the incoming beam with a 3.8 μm silica microsphere^14^. Also, these experimental results open a new method to improve the up-conversion emission intensity in biological samples with rare earth doped nanoparticles that can be used as nano-sensors. In another case, the generation of photocurrent in a commercial solar cell has been achieved under excitation at 1480 nm in fluoroindate glass samples co-doped with Er^3+^/Yb^3+^ ions^15^.

Fluoroindate glasses with their excellent spectroscopic properties offer the possibility of using these materials not only in the operation of erbium up-conversion lasers. Also, they belong to promising glass materials emitting near-infrared radiation. Nowadays there is a great interest in compact lasers operating in the near-infrared (1.5 μm) and mid-infrared (2.8 μm) for optical communications, medical and eye-safe light detecting and ranging applications. However, the near-infrared luminescence studies were limited practically to fluoroindate glasses containing Er^3+^ or Er^3+^/Yb^3+^ ions^10,13^. Luminescence spectra exhibit a highly intense signal at 1.5 μm due to ^4^I_13/2_ → ^4^I_15/2_ transition of Er^3+^. Decay measurements indicate that luminescence lifetime for the upper ^4^I_13/2_ laser state of Er^3+^ ions in fluoroindate glass is quite long and its value is close nearly to 10 ms at room temperature^10^. The mid-infrared luminescence results obtained for Er^3+^/Yb^3+^ ions in fluoroindate glass suggest that co-doping with Yb^3+^ favors only the up-conversion processes which depopulate efficiently the ^4^I_11/2_ state, reducing the emission intensity related to ^4^I_11/2_ → ^4^I_13/2_ transition of Er^3+^ ions at 2.8 μm^13^. These phenomena are important for diode-pumped solid-state lasers, which could provide a compact and efficient device with the advantage of easy coupling with fiber integrated optical systems. For diode-pumped lasers luminescence at near-infrared (1.5 μm) and mid-infrared (2.8 μm) spectral ranges, the trivalent Er^3+^ seems to be an excellent candidate due to ^4^I_13/2_ → ^4^I_15/2_ and ^4^I_11/2_ → ^4^I_13/2_ electronic transitions, respectively. From accumulated data it is quite well-known that the transmission capacity of WDM systems (wavelength division multiplexing) should be improved. Many research groups are looking for a new glass matrices and their fibers in order to obtain signal amplification beyond the conventional optical NIR window between 1530 and 1565 nm, commonly known as the C-band. In fact, spectral bandwidth for the commercial EDFA system (Erbium Doped Fiber Amplifiers) based on silicate glass is equal to 40 nm at C-band and broadband near-infrared transmission is limited, unfortunately^16^. For that reason, several glass matrices containing rare earth ions are still tested in order to obtain excellent materials with broader emission bands and longer lifetimes. These improved spectroscopic parameters are necessary for signal amplification in telecommunication and laser technology. Some recently published works for fluoroindate glass clearly indicate that a logical extension of Er^3+^-doped systems would be the addition of other rare earth ions such as Tm^3+^ or Ho^3+^ ions^17,18^. The interesting results for fluoroindate glasses co-doped with Ho^3+^/Tm^3+^ and Ho^3+^/Nd^3+^ ions^19,20^ emitting near-infrared and mid-infrared radiation at 2 μm and 3.9 μm under direct excitation (888 nm or 808 nm) have been also well presented and discussed in details. Among rare earths, the trivalent Pr^3+^ ions exhibit broadband near-infrared luminescence covering a wavelength range from 1.2 μm to 1.7 μm, which is really important for optical fiber amplifiers operating at O-band (1260–1360 nm), E-band (1360–1460 nm), S-band (1460–1530 nm), C-band (1530–1565 nm), and L-band (1565–1625 nm)^21^. The recent results indicate that glasses^22–25^ and crystals^26^ co-doped with Pr^3+^/Er^3+^ seems to be quite good candidates for broadband near-IR luminescence. The superbroadband near-IR luminescence is contributed mainly by the ^1^D_2_ → ^1^G_4_ (Pr^3+^) and ^4^I_13/2_ → ^4^I_15/2_ (Er^3+^) transitions which lead to emission lines located at about 1.48 and 1.53 μm^25^. To the best of our knowledge, these phenomena were not yet examined for fluoroindate glasses co-doped with Pr^3+^/Er^3+^.

## Results and discussion

### Fluoroindate glasses singly doped with Pr^3+^ and Er^3+^

**Figure 1:**
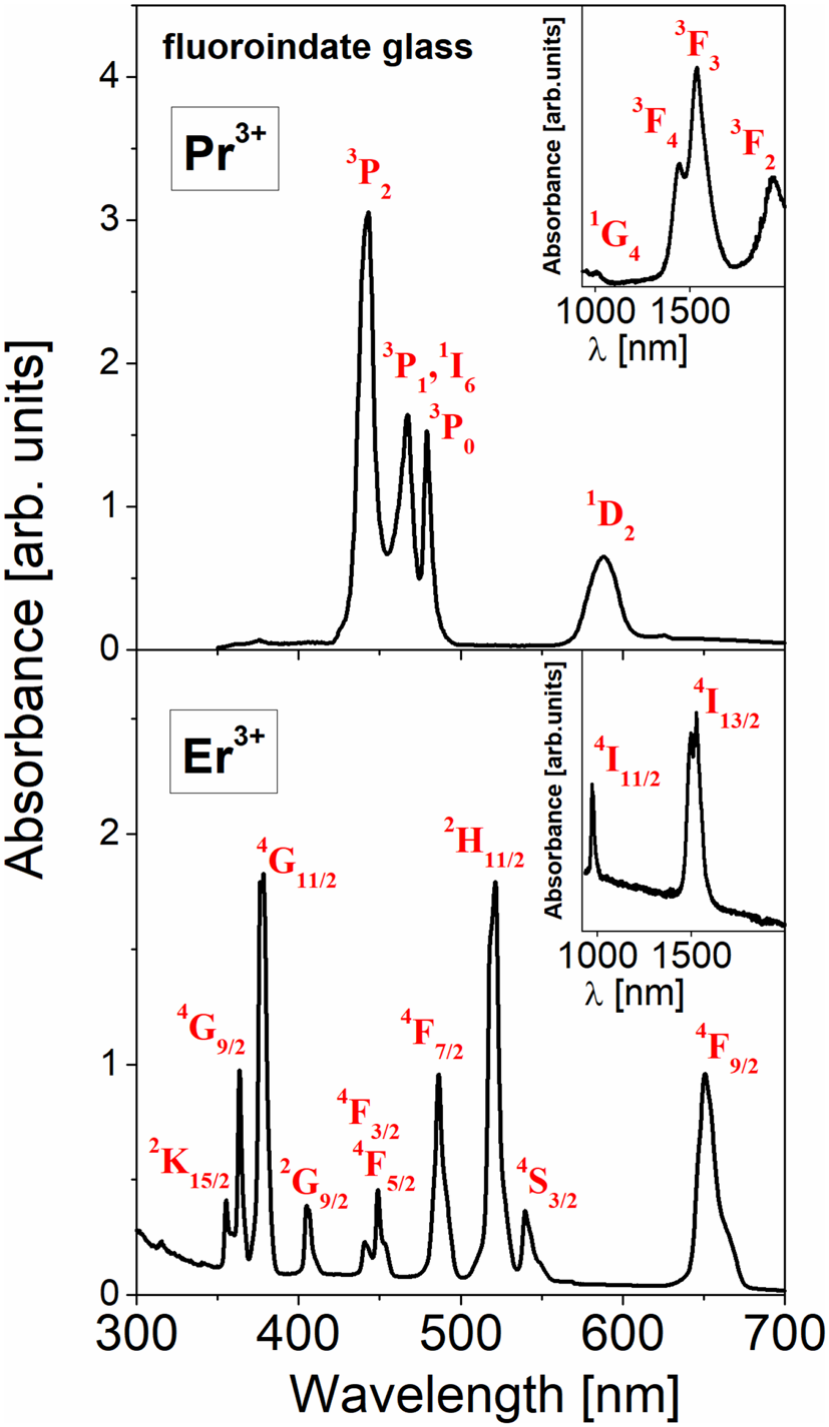
Absorption spectra of fluoroindate glasses singly doped with Pr^3+^ (top) and Er^3+^ (bottom).

The optical absorption spectra of fluoroindate glasses singly doped with Pr^3+^ and Er^3+^ ions were recorded at room temperature. They are presented in Fig. 1.

In general, the absorption bands are inhomogeneously broadened and clearly resolved, characteristic for 4f^2^ (Pr^3+^) and 4f^11^ (Er^3+^) transitions of rare earths. They are attributed to the electronic transitions originating from the ^3^H_4_ (Pr^3+^) and ^4^I_15/2_ (Er^3+^) ground states to the higher-lying excited states of rare earths. From the optical absorption spectra the experimental oscillator strengths have been determined. The band intensities are estimated by measuring the areas under the absorption lines using the equations:

1 $$P{}_{meas} = 4.318 \times 10^{ - 9} \int {\varepsilon (\nu )d\nu }$$

2 $$\varepsilon ({\text{n}}) \, = {\text{ A }}/{\text{ c l}}$$

where: *∫ε*(*ν*) represents the area under the absorption line, *A* indicates the absorbance, *c* is the concentration of the Ln^3+^ ion (Ln = Pr or Er) in mol l^−1^ and *l* denotes the optical path length. In the next step, the theoretical oscillator strengths for each transition of Pr^3+^ and Er^3+^ ions were calculated using the Judd–Ofelt theory^27,28^. The theoretical oscillator strength is defined as follows:

3 $$P_{calc} = \frac{{8\pi^{2} mc(n^{2} + 2)^{2} }}{3h\lambda (2J + 1) \cdot 9n} \times \sum\limits_{t = 2,4,6} {\Omega_{t} } ( < 4f^{N} J\left\| {U^{t} } \right\|4f^{N} J^{\prime} > )^{2}$$

where *m* is the mass of the electron, *c* is the velocity of light, *h* is the Planck constant and *λ* is the mean wavelength of the each transition. In order to perform the analysis, the refractive index of the medium (n = 1.48 for fluoroindate glass) and *U*^*t*^^*2*^ from Ref.^29^ representing the square of the matrix elements of the unit tensor operator *U*^*t*^ (connecting the initial J and final J' states) were used for calculations. Next, the experimental and theoretical oscillator strengths have been compared. They are listed in Table 1.

**Table 1:** Measured and calculated oscillator strengths for Pr^3+^ and Er^3+^ ions in fluoroindate glasses.

Ln^3+^	Levels	Average energy ν (cm^−1^)	Oscillator strengths (× 10^–6^)		
			P_meas_	P_calc_	Residuals
Pr^3+^	^3^H_6_,^3^F_2_	5000	3.688	3.689	0.001
	^3^F_4_,^3^F_3_	6700	8.850	8.865	0.015
	^1^G_4_	9900	0.160	0.265	0.105
	^1^D_2_	17,000	1.950	1.050	0.900
	^3^P_0_	20,900	2.400	2.950	0.550
	^3^P_1_,^1^I_6_	21,400	4.990	4.461	0.529
Er^3+^	^4^I_13/2_	6650	1.670	1.550	0.120
	^4^I_11/2_	10,200	0.550	0.650	0.100
	^4^F_9/2_	15,300	2.140	2.180	0.040
	^4^S_3/2_	18,200	0.510	0.610	0.100
	^2^H_11/2_	19,200	3.440	3.160	0.280
	^4^F_7/2_	20,500	1.870	2.370	0.500
	^4^F_5/2_,^4^F_3/2_	22,200	0.900	1.190	0.290
	^2^G_9/2_	24,600	0.660	0.900	0.240
	^4^G_11/2_	26,400	5.220	5.530	0.310
	^4^G_9/2_,^2^K_15/2_	27,750	2.190	1.840	0.350

Transitions are from the ^3^H_4_ (Pr^3+^) and ^4^I_15/2_ (Er^3+^) ground states to the levels indicated. The three phenomenological intensity parameters Ω_t_ (t = 2, 4, 6) are found to be (in 10^–20^ cm^2^ units): Ω_2_ = 2.01 ± 0.81, Ω_4_ = 5.25 ± 0.90, Ω_6_ = 5.10 ± 0.35, rms = 1.4 × 10^–6^ (for Pr^3+^) and Ω_2_ = 1.47 ± 0.22, Ω_4_ = 1.51 ± 0.30, Ω_6_ = 1.69 ± 0.11, rms = 0.7 × 10^–6^ (for Er^3+^), respectively.

The three phenomenological Judd–Ofelt intensity parameters *Ω*_*t*_ (t = 2, 4, 6) for rare earth ions in fluoroindate glasses are found to be (in 10^–20^ cm^2^ units); Ω_2_ = 2.01 ± 0.81, Ω_4_ = 5.25 ± 0.90, Ω_6_ = 5.10 ± 0.35, rms = 1.4 × 10^–6^ (for Pr^3+^) and Ω_2_ = 1.47 ± 0.22, Ω_4_ = 1.51 ± 0.30, Ω_6_ = 1.69 ± 0.11, rms = 0.7 × 10^–6^ (for Er^3+^). The fit quality was expressed by the magnitude of the root-mean-square deviation, which is defined by rms = Σ (P_meas_ – P_calc_)^2^.

In the next step, the intensity parameters *Ω*_*t*_ (t = 2, 4, 6) were applied to calculate the radiative transition rates, luminescence branching ratios and radiative lifetimes. The radiative transition rates *A*_*J*_ for excited levels of Pr^3+^ and Er^3+^ ions from an initial level *J* to a final ground level *J’* were calculated using the following relation:

4 $$A_{J} = \frac{{64\pi ^{4} e^{2} }}{{3h(2J + 1)\lambda ^{3} }} \times \frac{{n(n^{2} + 2)^{2} }}{9} \times \sum\limits_{{t = 2,4,6}} {\Omega _{t} } ( < 4f^{N} J\left\| {U^{t} } \right\|4f^{N} J' > )^{2}$$

The total radiative emission rate *A*_*T*_ involving all the intermediate terms is the sum of the *A*_*J*_ terms. The radiative lifetime *τ*_*rad*_ of an excited level is the inverse of the total radiative emission rate (Eq. 5), whereas the luminescence branching ratio *β* is due to the relative intensities of transitions from excited level to all terminal levels (Eq. 6).

5 $$\tau _{{rad}} = \frac{1}{{\sum\limits_{i} {A_{{Ji}} } }} = \frac{1}{{A_{T} }}$$

6 $$\beta = \frac{{A_{J} }}{{\sum\limits_{i} {A_{Ji} } }}$$

The calculated radiative transition rates A_J_, luminescence branching ratios β and corresponding radiative lifetimes τ_rad_ for Pr^3+^ and Er^3+^ ions in fluoroindate glasses are presented in Table 2. The calculation results are limited to transitions originating from the ^1^D_2_ and ^1^G_4_ excited levels of Pr^3+^ as well as the ^4^I_11/2_ and ^4^I_13/2_ excited levels of Er^3+^, from which the main luminescence lines in the near-infrared spectral range (950–1650 nm) occur.

**Table 2:** Calculated radiative transition rates A_J_, luminescence branching ratios β and corresponding radiative lifetimes τ_rad_ for Pr^3+^ and Er^3+^ ions in fluoroindate glasses.

Ln^3+^	transition	λ [nm]	A_J_ [s^-1^]	β	τ_rad_ [ms]	τ_m_ [ms]	η [%]
Pr^3+^	^1^D_2_–^3^H_4_	588	800	0.33	0.41	0.308	75
	^3^H_5_	680	14	0.01			
	^3^H_6_	805	300	0.12			
	^3^F_2_	845	540	0.22			
	^3^F_3_	950	75	0.03			
	^3^F_4_	995	480	0.20			
	^1^G_4_	1450	230	0.09			
	^1^G_4_–^3^H_4_	1010	24	0.07	2.80	0.365	13
	^3^H_5_	1335	232	0.65			
	^3^H_6_	1890	75	0.21			
	^3^F_2_	2110	1	< 0.01			
	^3^F_3_	2950	1	< 0.01			
	^3^F_4_	3400	24	0.07			
Er^3+^	^4^I_11/2_–^4^I_15/2_ ^4^I_13/2_	980 2817	134 15	0.90 0.10	6.71	6.65	99.1
	^4^I_13/2_–^4^I_15/2_	1535	116	1.00	8.62	8.60	99.8

The values of measured lifetimes τ_m_ and quantum efficiencies η for excited levels of Pr^3+^ and Er^3+^ ions are also indicated.

Measured lifetimes τ_m_ for the ^1^D_2_ and ^1^G_4_ (Pr^3+^) and the ^4^I_11/2_ and ^4^I_13/2_ (Er^3+^) excited levels are also given in Table 2 in order to determine quantum efficiencies η of rare earth ions in fluoroindate glasses using the following equation:

7 $$\eta = \frac{{\tau_{m} }}{{\tau_{rad} }} \times 100\%$$

**Figure 2:**
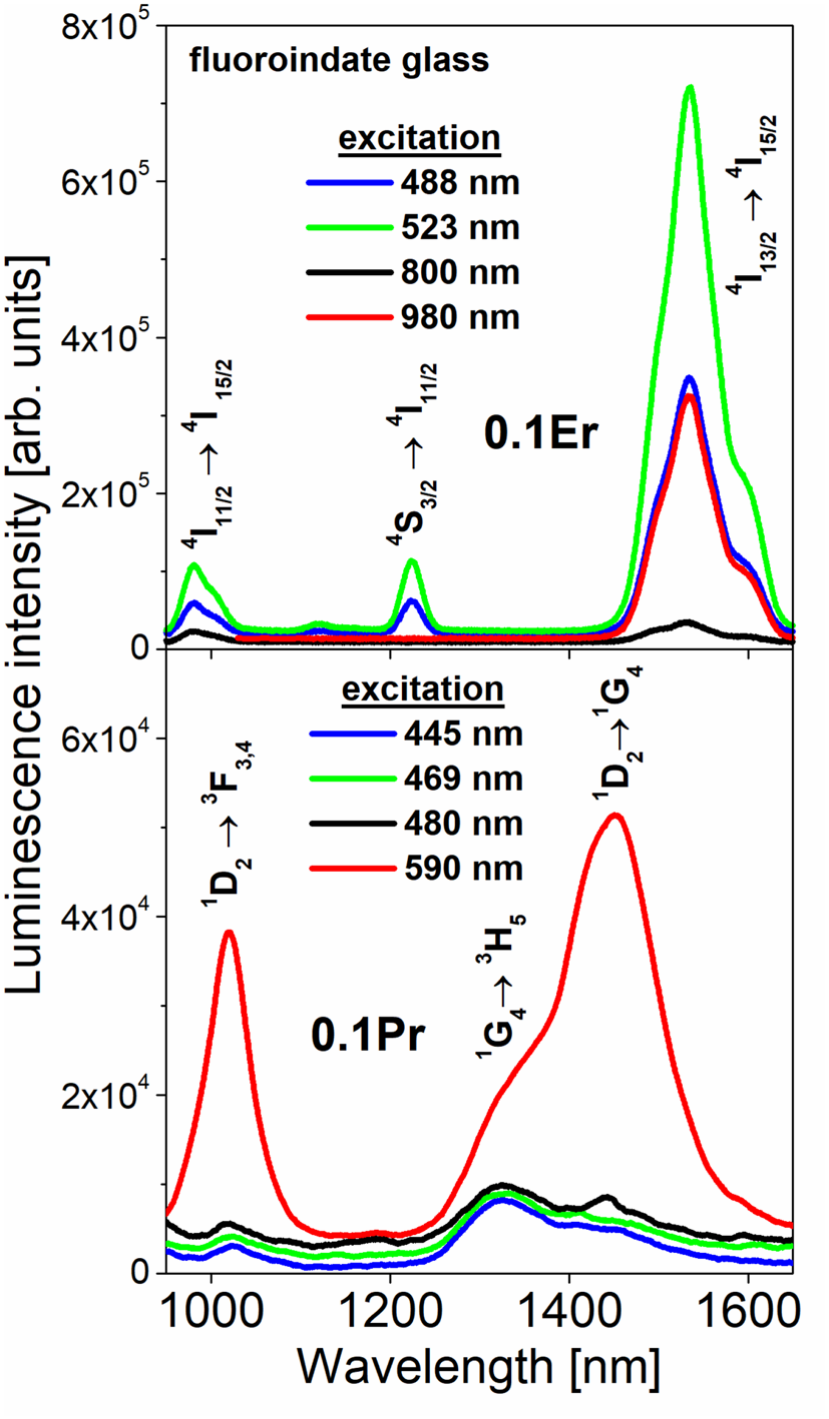
Near-infrared luminescence spectra of fluoroindate glasses doped with Pr^3+^ (top) and Er^3+^ (bottom).

Figure 2 presents near-infrared luminescence spectra of fluoroindate glasses singly doped with Er^3+^ and Pr^3+^. The spectra were measured for glass samples in the 950–1650 nm ranges. For both Er^3+^ and Pr^3+^ doped glass samples, the activator concentration is equal to 0.1 mol%.

For Er^3+^ singly doped glass, the spectra consist of luminescence bands centered at 980 nm and 1535 nm, which correspond to transitions originating from the ^4^I_11/2_ and ^4^I_13/2_ excited levels to the ^4^I_15/2_ ground level, respectively. When glass sample is excited at higher-lying ^4^F_7/2_ (488 nm) or ^2^H_11/2_ (523 nm) level, luminescence band near 1230 nm associated to the ^4^S_3/2_ → ^4^I_11/2_ (Er^3+^) transition is also well observed.

The most intense luminescence band is related to the main ^4^I_13/2_ → ^4^I_15/2_ near-infrared laser transition of Er^3+^. Its luminescence intensity depends strongly on excitation wavelengths. Several spectroscopic parameters for the ^4^I_13/2_ → ^4^I_15/2_ near-infrared transition of Er^3+^ ions in fluoroindate glass at 1535 nm, necessary for optical and laser characteristics, were determined. The luminescence linewidth defined as the full width at half maximum (FWHM) for the ^4^I_13/2_ → ^4^I_15/2_ (Er^3+^) transition is equal to 72 nm. The measured luminescence lifetime for the upper ^4^I_13/2_ laser level of Er^3+^ ions in fluoroindate glass is close to 8.6 ms, whereas radiative lifetime calculated from the Judd–Ofelt framework seems to be 8.62 ms (Table 2). Thus, the quantum efficiency for the upper ^4^I_13/2_ laser level of Er^3+^ ions in fluoroindate glass is nearly ~ 100%. The same situation is also observed for the higher-lying ^4^I_11/2_ level of Er^3+^, for which the measured and calculated radiative lifetimes and the quantum efficiency are close to τ_m_ = 6.65 ms, τ_rad_ = 6.71 ms and η =  ~ 99%. The quantum efficiency for the main ^4^I_13/2_ → ^4^I_15/2_ (Er^3+^) laser transition at 1535 nm in fluoroindate glass is significantly higher in comparison to the values obtained earlier for borate (η = 3%) and germanate (η = 71%) glasses^30^. This phenomenon is related directly to the lower phonon energy (hω = 510 cm^−1^) of the fluoroindate glass-host^17^.

The peak stimulated emission cross-section σ_em_ is one of the most important radiative parameters and should be also examined because the strong luminescence band at 1535 nm due to the ^4^I_13/2_ → ^4^I_15/2_ transition has been considered as a potential near-infrared laser emission of Er^3+^ ions in fluoroindate glass. It is generally accepted that an efficient laser transition is characterized by relatively large value of σ_em_. The peak stimulated emission cross-section σ_em_ can be obtained from the calculated radiative transition rate A_J_ using the following relation:

8 $$\sigma _{{em}} = \frac{{\lambda _{p}^{4} }}{{8\pi cn^{2} \Delta \lambda }}A_{J}$$

where λ_p_ is the peak luminescence wavelength, Δλ is the effective linewidth (FWHM), n is the refractive index and c is the velocity of light. The maximum peak stimulated emission cross-section is close to nearly 5.4 × 10^–21^ cm^2^ at 1535 nm for fluoroindate glass and its value is typical for fluoride laser glasses containing Er^3+^ ions^31^.

Near-infrared luminescence spectra of Pr^3+^ ions in fluoroindate glasses are also presented on Fig. 2 (bottom). In general, three near-infrared luminescence bands are quite well observed under different excitation wavelengths. They correspond to ^1^D_2_ → ^3^F_3,4_ (1050 nm), ^1^G_4_ → ^3^H_5_ (1335 nm) and ^1^D_2_ → ^1^G_4_ (1450 nm) electronic transitions of Pr^3+^. However, the intensities of luminescence bands of Pr^3+^ are extremely low, when glass sample was excited at higher lying ^3^P_2_ (445 nm), ^3^P_1_ (469 nm) or ^3^P_0_ (480 nm) level, respectively. In this case, the ^1^G_4_ → ^3^H_5_ transition with its FWHM value equal to about 195 nm is dominant transition of trivalent Pr^3+^. The ^1^G_4_ measured lifetime of Pr^3+^ (τ_m_ = 0.365 ms) is considerably lower than corresponding calculated radiative lifetime (τ_m_ = 2.8 ms). Although the luminescence branching ratio for the ^1^G_4_ → ^3^H_5_ transition at 1335 nm is relatively high and its value is close to *β* = 65%, the quantum efficiency (η = 13%) for the ^1^G_4_ excited level of Pr^3+^ ions is rather low (Table 2). Completely different situation is observed for Pr^3+^ ions in fluoroindate glass under direct excitation of ^1^D_2_ state at 590 nm. The near-infrared luminescence bands originating to transitions from the ^1^D_2_ level of Pr^3+^ ions are highly intense compared to the ^1^G_4_ → ^3^H_5_ transition at 1335 nm. In particular, broadband near-infrared spectra covering a wavelength range from 1300 nm to about 1650 nm are great of interest and really important for optical telecommunication^**21**^. The main most intense near-infrared luminescence band near 1450 nm is assigned to the ^1^D_2_ → ^1^G_4_ transition of Pr^3+^ and its value of FWHM is close to 130 nm. Although the luminescence branching ratio (*β* = 9%), the measured (τ_m_ = 0.308 ms) and calculated radiative (τ_m_ = 0.410 ms) lifetimes are not too high, the quantum efficiency for the ^1^D_2_ level of Pr^3+^ is close to η = 75% (Table 2). Furthermore, the peak stimulated emission cross-section for the ^1^D_2_ → ^1^G_4_ transition of Pr^3+^ ions in fluoroindate glass was determined. From literature data it is well-known that inorganic glasses singly doped with praseodymium exhibit fascinating prospects in broadband near-infrared fiber amplifier, when the emission cross-section profile is large^21^. In our case, the peak stimulated emission cross-section for the ^1^D_2_ → ^1^G_4_ transition of Pr^3+^ ions is also relatively large. Its value is close to 0.5 × 10^−20^cm^2^. Finally, the stimulated emission cross-section (σ_em_), the emission linewidth (FWHM) and the measured lifetime (τ_m_) were applied to calculate the gain bandwidth (σ_em_ × FWHM product) and the figure of merit (FOM) given by σ_em_ × τ_m_, respectively. It is interesting to see that the σ_em_ × FWHM product for the ^1^D_2_ → ^1^G_4_ transition of Pr^3+^ ions in fluoroindate glass is equal to 65 × 10^−27^ cm^3^, which is rather low compared to the following values: 176.4 × 10^−27^ cm^3^ for fluorotellurite glass^**32**^ and 203.5 × 10^−27^ cm^3^ for gallo-germanate glass with BaF_2_^33^. This behavior is mainly due to the fact that luminescence linewidth for the ^1^D_2_ → ^1^G_4_ transition of Pr^3+^ ions in fluoroindate glass (FWHM = 130 nm) is considerably lower than values obtained for fluorotellurite glass (FWHM = 196 nm) and gallo-germanate glass (FWHM = 208.5 nm). On the other hand, the figure of merit (FOM) for the ^1^D_2_ → ^1^G_4_ transition of Pr^3+^ ions in fluoroindate glass is relatively larger (σ_em_ x τ_m_ = 154 × 10^−26^ cm^2^s) in comparison to the values 83.1 × 10^-26^cm^2^s for fluorotellurite glass^32^ and 107.5 × 10^−26^ cm^2^s for gallo-germanate glass modified by BaF_2_^33^. Our experimental studies and theoretical calculations demonstrate that Pr^3+^ singly doped fluoroindate glasses are promising for broadband near-infrared amplifiers.

### Fluoroindate glasses co-doped with Pr^3+^/Er^3+^

**Figure 3:**
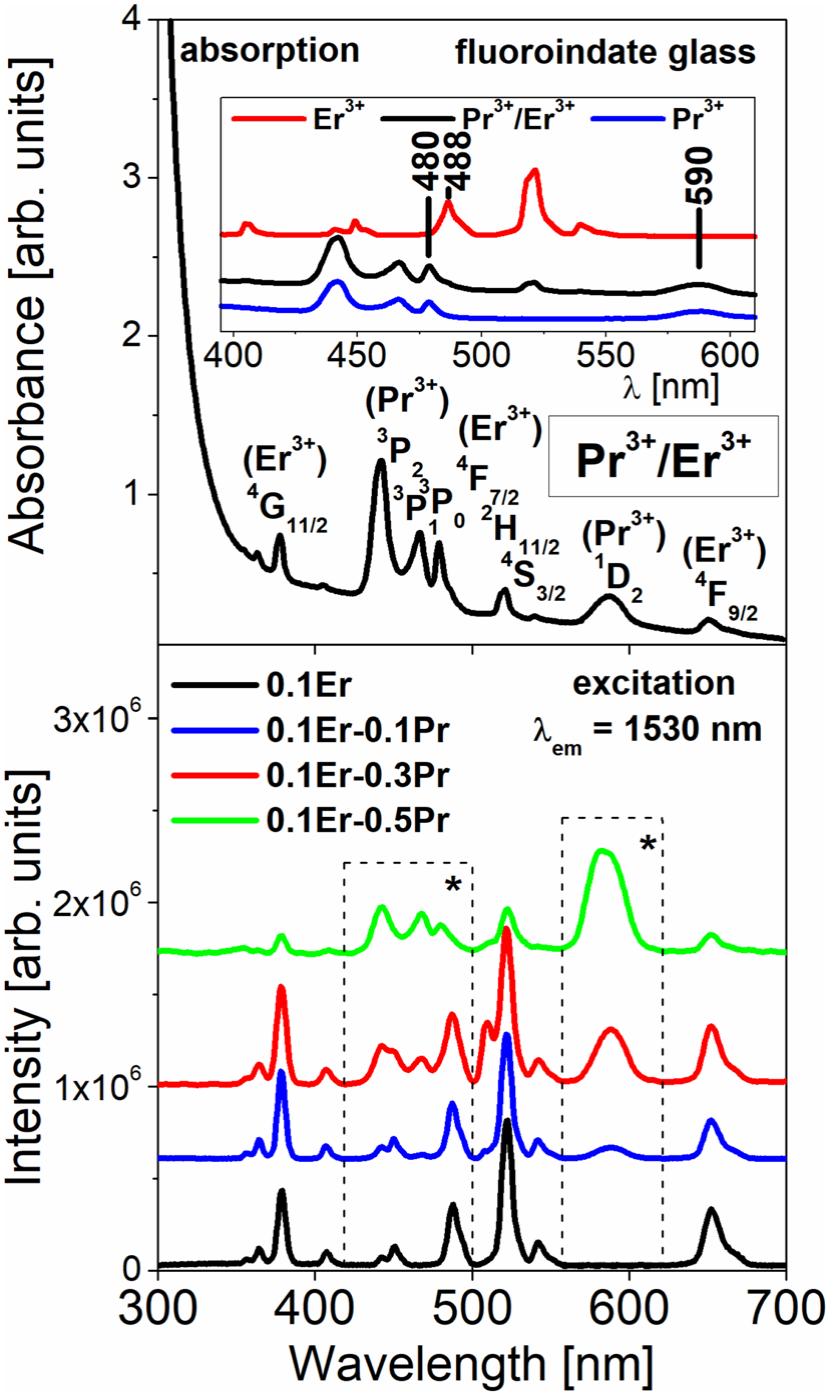
Absorption (top) and excitation (bottom) spectra of fluoroindate glasses co-doped with Pr^3+^/Er^3+^. The results are compared to the spectra, which were measured for rare earth singly doped glass samples.

Figure 3 present absorption (top) and excitation (bottom) spectra of fluoroindate glasses co-doped with Pr^3+^/Er^3+^. The results are compared to the absorption spectra, which were measured for rare earth singly doped glass samples (inset).

The absorption spectrum for Pr^3+^/Er^3+^ co-doped glass sample consists of several bands corresponding to both electronic transitions of rare earths originating from the ground levels ^3^H_4_ (Pr^3+^) and ^4^I_15/2_ (Er^3+^) to the excited levels. In particular, two spectral ranges near 590 nm and between 480 and 488 nm are important from the spectroscopic point of view. In the first spectral range near 590 nm, the absorption band associated to ^3^H_4_ → ^1^D_2_ transition of Pr^3+^ is located. In the second spectral range, two absorption bands related to ^3^H_4_ → ^3^P_0_ transition of Pr^3+^ (480 nm) and ^4^I_15/2_ → ^4^F_7/2_ transition of Er^3+^ (488 nm) are quite well overlapped.

The excitation spectra measured for Pr^3+^/Er^3+^ co-doped glass samples under monitoring emission wavelength 1530 nm (^4^I_13/2_ Er^3+^) give interesting results. It is clearly shown in these two spectral ranges denoted as (*) that the intensity of band at 590 nm assigned to ^3^H_4_ → ^3^P_0_ transition of Pr^3+^ ions increase, whereas the relative band intensities of ^3^H_4_ → ^3^P_0_ (Pr^3+^) and ^4^I_15/2_ → ^4^F_7/2_ (Er^3+^) transitions located between 480 and 488 nm are drastically changed with increasing Pr^3+^ concentration. It also suggests that the energy transfer process between Pr^3+^ and Er^3+^ ions in fluoroindate glass occurs.

**Figure 4:**
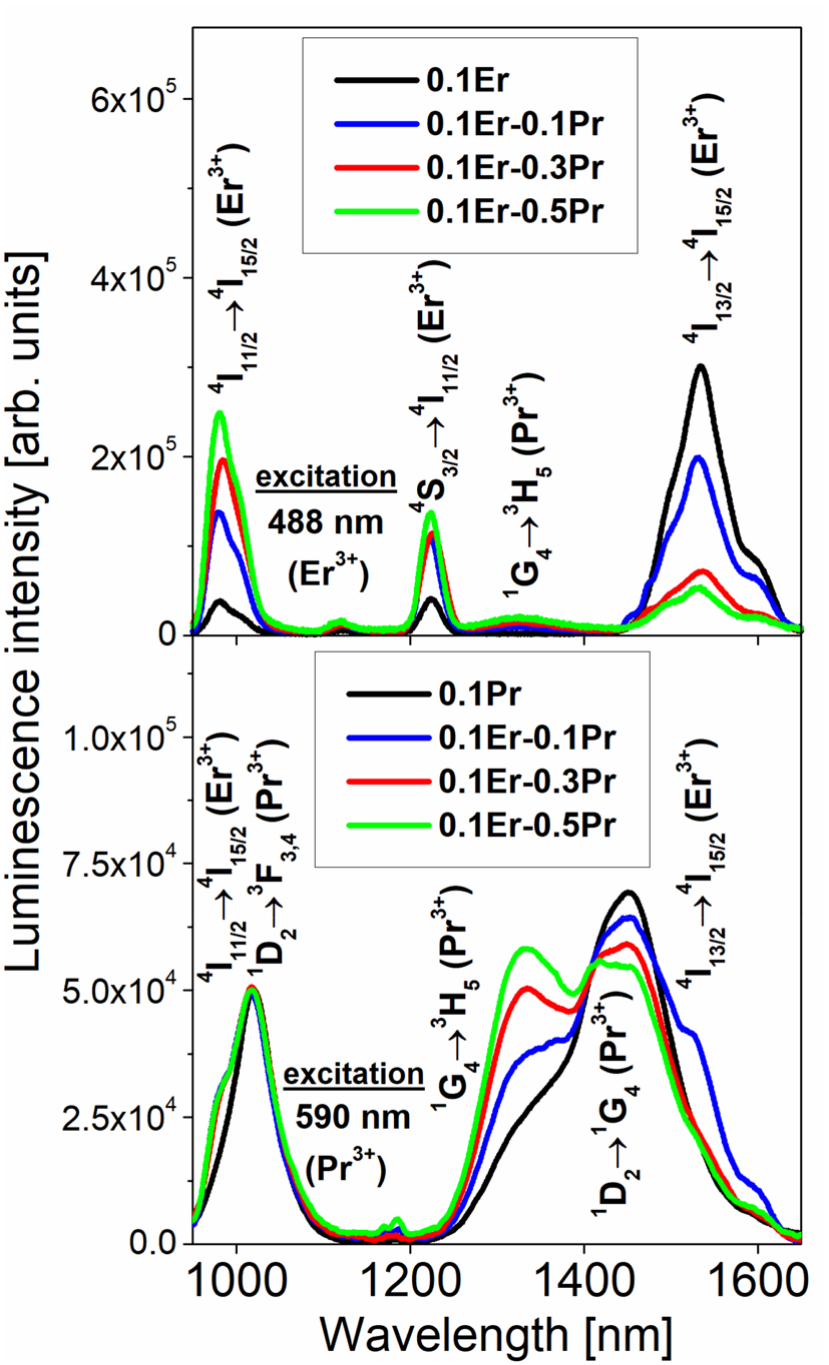
Near-infrared luminescence spectra of fluoroindate glasses co-doped with Pr^3+^/Er^3+^ ions under direct 488 nm (top) and 590 nm (bottom) excitation. The results are compared to the spectra, which were measured for rare earth singly doped glass samples.

In the next step, luminescence spectra of fluoroindate glasses co-doped with Pr^3+^/Er^3+^ have been examined under different excitation wavelengths. In general, Pr^3+^/Er^3+^ co-doped glasses belong to amorphous systems emitting visible light and near-infrared radiation. It is quite well-known that several visible emission bands from the ^3^P_0_, ^1^D_2_ (Pr^3+^) and the (^2^H_11/2_,^4^S_3/2_), ^4^F_9/2_ excited levels of rare earth ions can be well observed for Pr^3+^/Er^3+^ co-doped glasses^34^. These aspects for fluoroindate glass are not presented and discussed here. Our investigations are limited to Pr^3+^/Er^3+^ co-doped fluoroindate glass emitting near-infrared radiation. Figure 4 shows near-infrared luminescence spectra of fluoroindate glasses co-doped with Pr^3+^/Er^3+^ under direct 488 nm (top) and 590 nm (bottom) excitation. The results are compared to the spectra, which were measured for rare earth singly doped glass samples.

**Figure 5:**
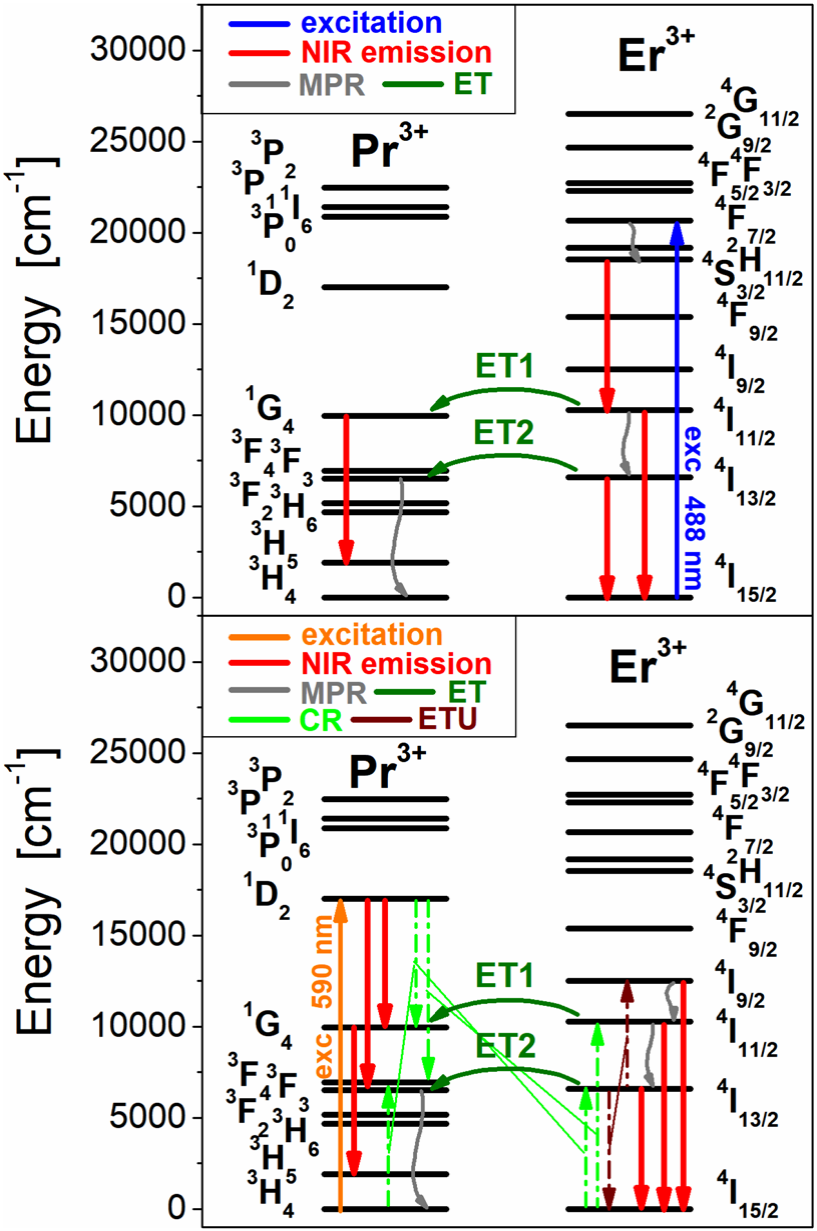
Energy level scheme for fluoroindate glass co-doped with Pr^3+^/Er^3+^. All transitions are also indicated for glass samples excited at 488 nm (top) and 590 nm (bottom).

Four emission bands at 980 nm, 1230 nm, 1335 nm and 1535 nm are observed for Pr^3+^/Er^3+^ co-doped fluoroindate glasses under direct excitation of ^4^F_7/2_ level of Er^3+^ at 488 nm. They correspond to ^4^I_11/2_ → ^4^I_15/2_ (Er^3+^), ^4^S_3/2_ → ^4^I_11/2_ (Er^3+^), ^1^G_4_ → ^3^H_5_ (Pr^3+^) and ^4^I_13/2_ → ^4^I_15/2_ (Er^3+^) transitions, respectively. In contrast to Er^3+^ singly doped glass, the intensity of emission band at 1535 nm due to the main ^4^I_13/2_ → ^4^I_15/2_ (Er^3+^) near-infrared laser transition is reduced, whereas the emission band intensities related to ^4^I_11/2_ → ^4^I_15/2_ and ^4^S_3/2_ → ^4^I_11/2_ (Er^3+^) transitions increase with increasing Pr^3+^ concentration in samples co-doped with Pr^3+^/Er^3+^. The emission linewidth for ^4^I_13/2_ → ^4^I_15/2_ (Er^3+^) near-infrared laser transition is nearly independent on Pr^3+^ concentration and its FWHM value is close to 75 ± 3 nm. The intensity of emission band at 1335 nm associated to the ^1^G_4_ → ^3^H_5_ (Pr^3+^) transition increase with increasing activator (Pr^3+^) concentration. The experimental results for Pr^3+^/Er^3+^ co-doped fluoroindate glasses clearly suggest (a) the presence of the energy transfer process from Er^3+^ to Pr^3+^ and (b) near-infrared emission of Pr^3+^ at 1335 nm under direct excitation of Er^3+^. Further investigations indicate that five near-infrared luminescence bands at about 980 nm, 1050 nm, 1335 nm, 1450 nm and 1535 nm are quite well observed for Pr^3+^/Er^3+^ co-doped fluoroindate glasses under direct excitation of ^1^D_2_ level of Pr^3+^ at 590 nm. Emission bands are due to the ^4^I_11/2_ → ^4^I_15/2_ (Er^3+^), ^1^D_2_ → ^3^F_3,4_ (Pr^3+^), ^1^G_4_ → ^3^H_5_ (Pr^3+^), ^1^D_2_ → ^1^G_4_ (Pr^3+^) and ^4^I_13/2_ → ^4^I_15/2_ (Er^3+^), respectively. The presence of ^4^I_11/2_ → ^4^I_15/2_ and ^4^I_13/2_ → ^4^I_15/2_ transitions of Er^3+^ in the luminescence spectra measured under direct excitation of Pr^3+^ confirms the energy transfer process from Pr^3+^ to Er^3+^ ions in fluoroindate glasses co-doped with Pr^3+^/Er^3+^. Firstly, broadband near-infrared luminescence at around 1000 nm in Pr^3+^/Er^3+^ co-doped glass samples is due to two overlapped ^4^I_11/2_ → ^4^I_15/2_ (Er^3+^) and ^1^D_2_ → ^3^F_3,4_ (Pr^3+^) transitions. These phenomena were studied previously for fluorotellurite glass co-doped with Pr^3+^/Er^3+^ ions^35^. Secondly, the near-infrared emission band at 1535 nm due to ^4^I_13/2_ → ^4^I_15/2_ (Er^3+^) transition is overlapped with ^1^G_4_ → ^3^H_5_ transition of Pr^3+^ in glass samples co-doped with Pr^3+^/Er^3+^. These effects are not observed for Pr^3+^ singly doped glass sample. Furthermore, the intensity of emission band at 1335 nm due to the ^1^G_4_ → ^3^H_5_ (Pr^3+^) transition enhance, whereas the intensity of emission band at 1450 nm corresponding to ^1^D_2_ → ^1^G_4_ (Pr^3+^) is reduced with increasing Pr^3+^ concentration in glass composition. Interestingly, luminescence linewidth for broadband near-infrared luminescence covering a wavelength range from 1300 nm to about 1650 nm and associated to two overlapped ^1^G_4_ → ^3^H_5_ and ^1^D_2_ → ^1^G_4_ transitions of Pr^3+^ enhance drastically in fluoroindate glasses co-doped with Pr^3+^/Er^3+^. In contrast to Pr^3+^ singly doped glass (FWHM = 130 nm), emission linewidth for glass samples co-doped with Pr^3+^/Er^3+^ increase to nearly 225 ± 5 nm and its value slightly depends on Pr^3+^ concentration. All near-infrared transitions indicated for Pr^3+^/Er^3+^ co-doped fluoroindate glasses excited directly at 488 nm or 590 nm are schematized on the energy level diagram (Fig. 5).

The mechanisms for Pr^3+^/Er^3+^ co-doped glass system involving possible energy transfer and radiative/nonradiative relaxation channels, i.e. energy transfer routes (ET), multiphonon relaxation (MPR) and cross-relaxation (CR) processes, have been proposed and discussed in details by Zhou et al.^25^.

When glass sample co-doped with Pr^3+^/Er^3+^ is directly pumped at 488 nm, the excited level ^4^F_7/2_ (Er^3+^) is well populated by the ground-state absorption process (GSA). The energy transfer process (ET) from the ^4^F_7/2_ (Er^3+^) level to the ^3^P_0_ (Pr^3+^) level is not observed and it is rather impossible, because the ^3^P_0_ level of Pr^3+^ is higher-lying than the ^4^F_7/2_ level of Er^3+^. There is also the main reason that near-infrared luminescence bands from the higher-lying ^1^D_2_ level of Pr^3+^ ions in fluoroindate glasses are not observed under 488 nm excitation of Er^3+^. The excitation energy transfers very fast nonradiatively to the ^4^S_3/2_ level by multiphonon relaxation (MPR) and then relaxes radiatively generating near-infrared emission at 1230 nm associated to the ^4^S_3/2_ → ^4^I_11/2_ transition of Er^3+^. The another possible way to depopulate ^4^F_7/2_ level is nonradiative relaxation to the ^4^I_11/2_ level by MPR process owing to small energy gaps between the ^4^F_7/2_ excited level and lower-lying levels or cross-relaxation process (CR): [^4^F_7/2_, ^4^I_15/2_ → ^4^I_11/2_, ^4^I_11/2_], when concentration of Er^3+^ ions in glass sample is relatively high. Next, part of excitation energy is transferred nonradiatively from the ^4^I_11/2_ level to the lower-lying ^4^I_13/2_ level by MPR process, and consequently two near-infrared luminescence bands centered at 980 nm and 1535 nm due to ^4^I_11/2_ → ^4^I_15/2_ and ^4^I_13/2_ → ^4^I_15/2_ transitions are well observed. Finally, the excitation energy is successfully transferred from the ^4^I_11/2_ (Er^3+^) level to the ^1^G_4_ (Pr^3+^) level (ET1) and the ^4^I_13/2_ (Er^3+^) level to the ^3^F_3,4_ (Pr^3+^) level (ET2) by energy transfer processes. In particular, the ET1 process plays the important role, leading to near-infrared luminescence at 1335 nm due to ^1^G_4_ → ^3^H_5_ transition of Pr^3+^ under direct excitation of Er^3+^.

When glass sample co-doped with Pr^3+^/Er^3+^ is directly pumped at 590 nm, the excited level ^1^D_2_ (Pr^3+^) is well populated by the ground-state absorption process (GSA). Part of excitation energy relaxes radiatively and two near-infrared luminescence bands at about 1050 nm and 1450 nm are observed, which correspond to transitions originating from the ^1^D_2_ excited level to the lower-lying ^3^F_3,4_ and ^1^G_4_ levels of Pr^3+^. Another part of excitation energy is transferred nonradiatively by well-known CR process: [^1^D_2_, ^3^H_4_ → ^1^G_4_, ^3^F_3,4_]^36^. At this moment, it should be also pointed that the second CR process [^1^D_2_, ^3^H_4_ → ^3^F_3,4_, ^1^G_4_] was also proposed, but it is still not sure which one is more dominant. For Pr^3+^/Er^3+^ co-doped samples, another cross-relaxation route that depopulate efficiently the ^1^D_2_ (Pr^3+^) level is also possible. This can be attributed to the following CR process: [^1^D_2_ (Pr^3+^), ^4^I_15/2_ (Er^3+^) → ^1^G_4_ (Pr^3+^), ^4^I_13/2_ (Er^3+^)]. Thus, part of the excitation energy is transferred from Pr^3+^ to Er^3+^ ions, giving important contribution to near-infrared emission at 1535 nm related to ^4^I_13/2_ → ^4^I_15/2_ transition of Er^3+^. However, the near-infrared luminescence at 980 nm due to ^4^I_11/2_ → ^4^I_15/2_ transition of Er^3+^ is also successfully observed under excitation of Pr^3+^ ions at 590 nm (Fig. 4). The back energy transfer process from the ^1^G_4_ (Pr^3+^) level to the ^4^I_11/2_ (Er^3+^) level is rather impossible, because the ^4^I_11/2_ level of Er^3+^ is higher-lying than the ^1^G_4_ level of Pr^3+^.

Based on the energy level diagram, the ^4^I_11/2_ level of Er^3+^ ions in Pr^3+^/Er^3+^ co-doped glass samples can be populated in two ways. First, the presence of the phonon-assisted energy transfer process from the ^1^D_2_ (Pr^3+^) level to the ^4^F_9/2_ (Er^3+^) is proposed but it should be ignored considering the relatively large energy gap between them (~ 1500 cm^−1^) and the absence of luminescence lines from the ^4^F_9/2_ level^**25**^, for example well-known ^4^F_9/2_ → ^4^I_15/2_ red transition of Er^3+^ ions at 670 nm. The second way is the possible cross-relaxation process: [^1^D_2_ (Pr^3+^), ^4^I_15/2_ (Er^3+^) → ^3^F_3,4_ (Pr^3+^), ^4^I_11/2_ (Er^3+^)]. The question how higher-lying ^4^I_11/2_ level of Er^3+^ is populated under direct 590 nm excitation of Pr^3+^ is still open and actual, and further experiments are necessary to understand the population mechanism and multichannel relaxation in Pr^3+^/Er^3+^ co-doped glass systems. The presence of cross-relaxation processes from the ^1^D_2_ (Pr^3+^) level in fluoroindate glasses co-doped with Pr^3+^/Er^3+^ was confirmed by luminescence spectra and decay measurements.

**Figure 6:**
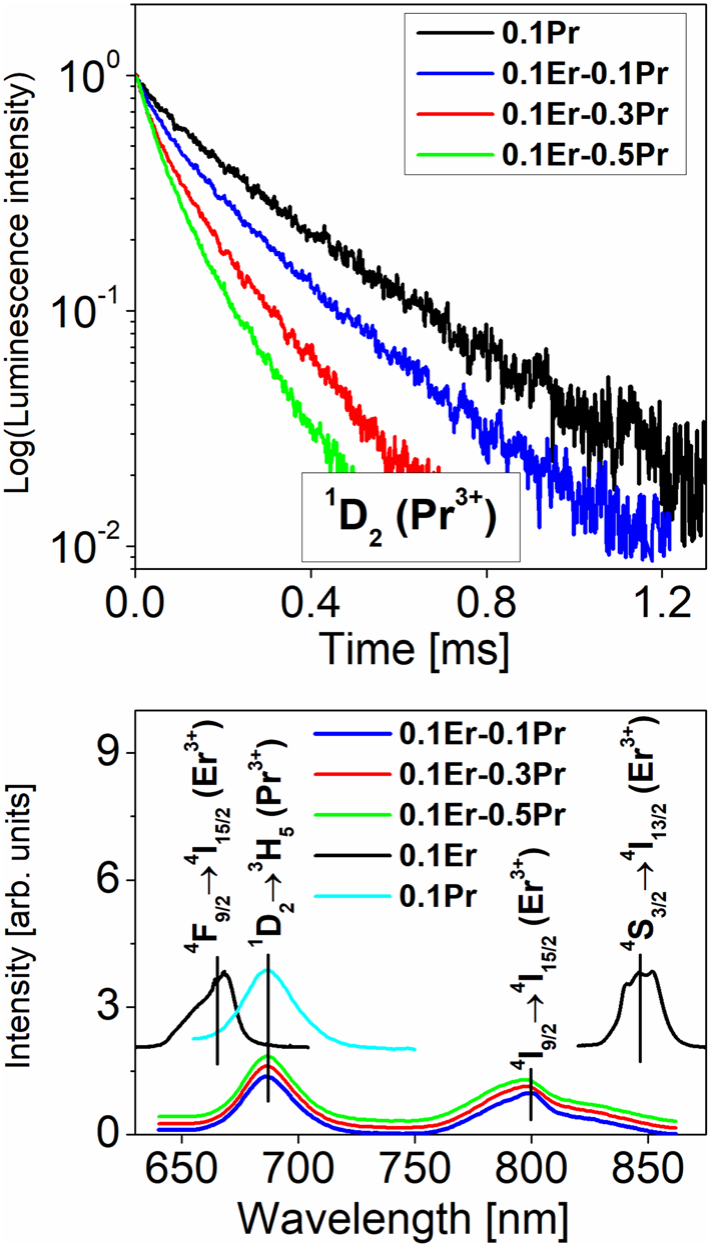
Luminescence decays from ^1^D_2_ (Pr^3+^) excited state (top) and visible-NIR emission spectra (bottom) of fluoroindate glasses containing Pr^3+^, Er^3+^ and Pr^3+^/Er^3+^.

Luminescence decays from the ^1^D_2_ level of Pr^3+^ ions in fluoroindate glasses co-doped with Pr^3+^/Er^3+^ are presented in Fig. 6 (top). The luminescence decay curves for the ^1^D_2_ level of Pr^3+^ were measured under monitoring emission wavelength 1450 nm.

Based on decays, luminescence lifetimes for the ^1^D_2_ level of Pr^3+^ were determined. For Pr^3+^/Er^3+^ co-doped glass samples, the ^1^D_2_ luminescence lifetime is reduced from 197 µs (0.1Er-0.1Pr) to 127 µs (0.1Er-0.3Pr) and 92 µs (0.1Er-0.5Pr) with increasing Pr^3+^ concentration. This behavior may be attributed to the presence of cross-relaxation processes between neighboring pairs Pr^3+^–Pr^3+^ and Pr^3+^–Er^3+^ in fluoroindate glasses. Also, we compare results for Pr^3+^-doped fluoroindate glasses in the absence and presence of Er^3+^. Thus, the measured ^1^D_2_ lifetime is reduced from 308 µs (0.1Pr) to 197 µs (0.1Er–0.1Pr), whereas the quantum efficiency for the ^1^D_2_ excited level of Pr^3+^ decreases from 75% (0.1Pr) to 48% (0.1Er–0.1Pr).

Figure 6 (bottom) shows the visible-NIR emission spectra of fluoroindate glasses singly doped with Pr^3+^ and Er^3+^ and doubly doped with Pr^3+^/Er^3+^. In the 630–880 nm spectral range, two emission bands are well observed for Er^3+^ singly doped glass under 488 nm excitation. They correspond to the ^4^F_9/2_ → ^4^I_15/2_ red transition near 670 nm and the ^4^S_3/2_ → ^4^I_13/2_ NIR transition at about 840 nm. The ^4^F_9/2_ → ^4^I_15/2_ red transition at 670 nm is not observed for Pr^3+^/Er^3+^ co-doped glass samples under direct excitation of Pr^3+^ at 590 nm. It was also verified for fluorotellurite glass co-doped with Pr^3+^/Er^3+^ excited at 594 nm^25^. In comparison to the results obtained for Pr^3+^ singly doped glass, the emission band at about 690 nm related to the ^1^D_2_ → ^3^H_5_ (Pr^3+^) transition was registered in this spectral range for Pr^3+^/Er^3+^ co-doped glass samples. Unexpectedly, the additional NIR luminescence band at 800 nm corresponding to the ^4^I_9/2_ → ^4^I_15/2_ transition of Er^3+^ ions was also detected in Pr^3+^/Er^3+^ co-doped fluoroindate glasses under direct excitation ^1^D_2_ (Pr^3+^) level at 590 nm. For that reason, it is interesting to explain how the ^4^I_9/2_ (Er^3+^) level is populated in Pr^3+^/Er^3+^ co-doped glass samples excited at the ^1^D_2_ (Pr^3+^) level. We postulate the following mechanisms involving cross-relaxation (CR) and energy transfer up-conversion (ETU) processes applied to populate ^4^I_9/2_, ^4^I_11/2_ and ^4^I_13/2_ excited levels of Er^3+^. The excited level ^1^D_2_ (Pr^3+^) is depopulated through the cross-relaxation processes: [^1^D_2_ (Pr^3+^), ^4^I_15/2_ (Er^3+^) → ^1^G_4_ (Pr^3+^), ^4^I_13/2_ (Er^3+^)] and [^1^D_2_ (Pr^3+^), ^4^I_15/2_ (Er^3+^) → ^3^F_3,4_ (Pr^3+^), ^4^I_11/2_ (Er^3+^)]. Thus, both ^4^I_11/2_ and ^4^I_13/2_ levels are well populated and near-infrared emission bands at 980 nm and 1535 nm due to the ^4^I_11/2_ → ^4^I_15/2_ and ^4^I_13/2_ → ^4^I_15/2_ transitions of Er^3+^ occur. Additionally, other processes play significant role in changing the population of ^4^I_11/2_ and ^4^I_13/2_ excited levels of Er^3+^ ions. The energy transfer up-conversion process (ETU): [^4^I_13/2_, ^4^I_13/2_ → ^4^I_9/2_, ^4^I_15/2_] gives important contribution to efficient population of higher-lying ^4^I_9/2_ level of Er^3+^ ions^37^. Further studies for the Er^3+^/Tm^3+^/Pr^3+^ triply doped fluoride glass indicate that the ETU process [^4^I_13/2_, ^4^I_13/2_ → ^4^I_9/2_, ^4^I_15/2_] increases population of the ^4^I_9/2_ level and then the lower-lying ^4^I_11/2_ level can be populated by a fast multiphonon relaxation from the ^4^I_9/2_ level, which leads to an increase of mid-infrared emission at 2700 nm^38^. Owing to presence of ETU process, it is possible to detect near-infrared emission peaking at 800 nm for Pr^3+^/Er^3+^ co-doped fluoroindate glass, which corresponds to the ^4^I_9/2_ → ^4^I_15/2_ transition of Er^3+^. The origin of near-infrared luminescence of Er^3+^ at 800 nm has been examined in an excellent work published recently^39^. It was stated that there is no NIR emission found at 800 nm, when the samples are excited to the higher-lying levels (^2^H_11/2_ or ^4^F_9/2_) than the ^4^I_9/2_ level, and the nonradiative relaxation from the upper excited levels to the ^4^I_9/2_ emitting level of Er^3+^ ions is extremely inefficient. The ^4^I_9/2_ level of Er^3+^ is mainly populated by the adjacent lower-lying ^4^I_11/2_ level upon excitation at 980 nm^39^. It was also confirmed for Er^3+^ doped chalcogenide glasses and fibers, where the conversion of incoherent infrared light around 4400 nm into a near-infrared signal at 810 nm was obtained by simultaneously 982 nm pumping^40^.

Further spectroscopic analysis of Pr^3+^/Er^3+^ co-doped fluoroindate glasses excited at 488 nm (Fig. 4) clearly indicate that the intensity of near-infrared luminescence band at 1535 nm due to the main ^4^I_13/2_ → ^4^I_15/2_ (Er^3+^) laser transition is decreased with increasing Pr^3+^ concentration in comparison to the intensities of ^4^I_11/2_ → ^4^I_15/2_ and ^4^S_3/2_ → ^4^I_11/2_ (Er^3+^) transitions of Er^3+^. Zhou et al.^25^ suggested that the relative increase of the near-infrared luminescence of Er^3+^ at 1230 nm compared with of 1530 nm can be ascribed to the improved population inversion between the upper and lower levels. Moreover, the decrease of near-infrared luminescence at 1530 nm is due to the energy transfer process from the ^4^I_13/2_ (Er^3+^) level to the ^3^F_3,4_ (Pr^3+^) levels. At a consequence, near-infrared emission bands related to transitions originating from the ^4^I_11/2_ level to the lower-lying ^4^I_13/2_ (2700 nm) and ^4^I_15/2_ (980 nm) levels can be enhanced. The luminescence decay analysis for the ^4^I_11/2_ and ^4^I_13/2_ excited levels of Er^3+^ ions in Pr^3+^/Er^3+^ co-doped fluoroindate glasses confirms this hypothesis.

**Figure 7:**
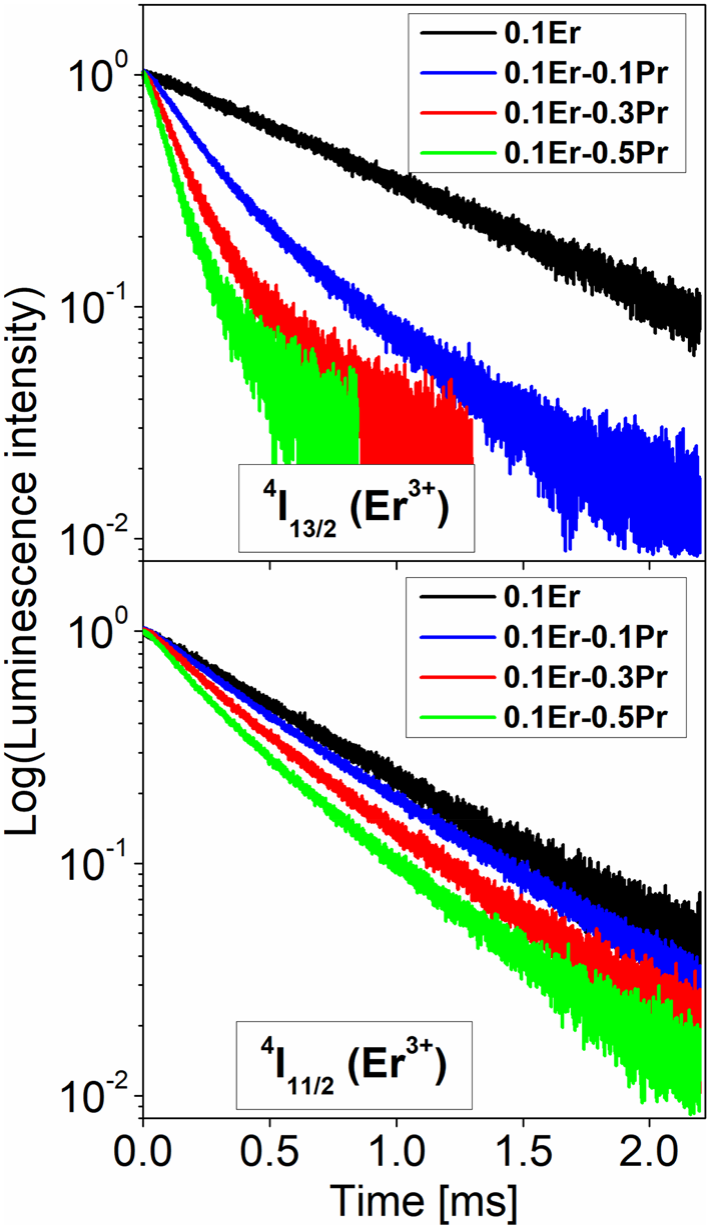
Luminescence decays from ^4^I_13/2_ (top) and ^4^I_11/2_ (bottom) excited states of Er^3+^ ions in fluoroindate glasses co-doped with Pr^3+^/Er^3+^.

**Figure 8:**
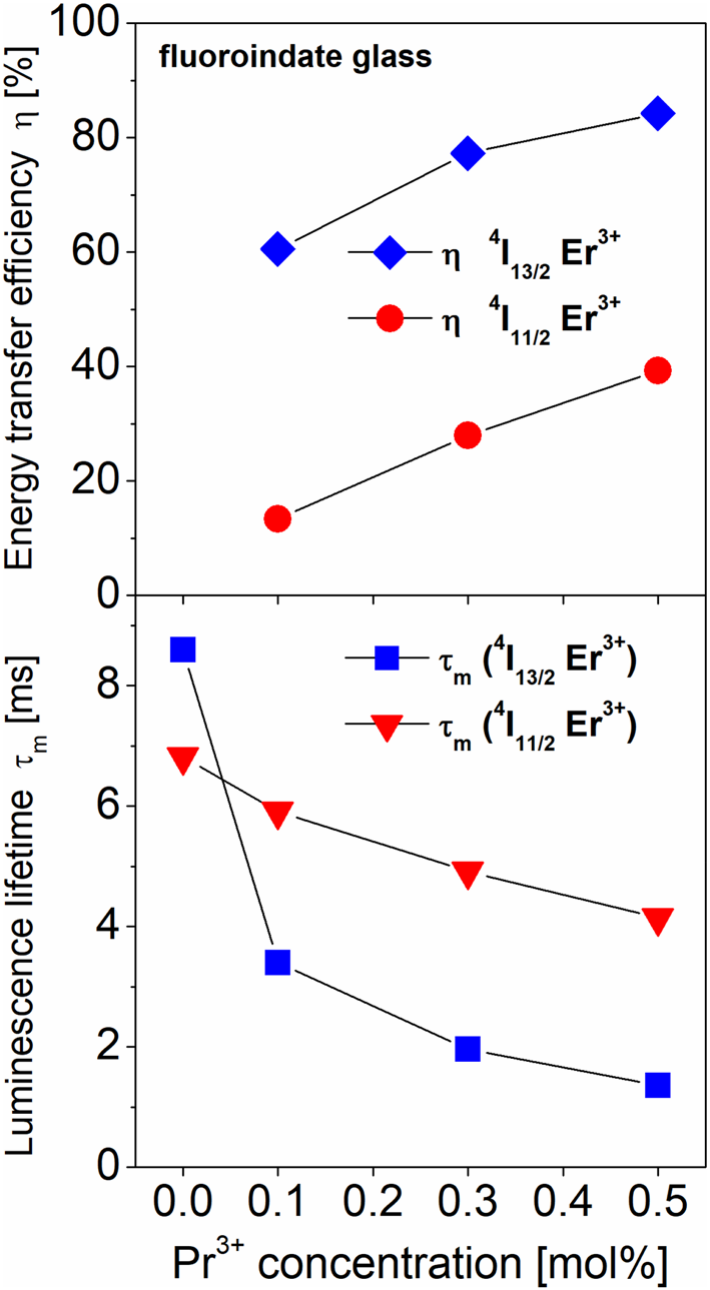
Luminescence lifetimes and energy transfer efficiencies varying with Pr^3+^ concentration.

Figure 7 shows luminescence decay curves for the ^4^I_13/2_ and ^4^I_11/2_ excited states of Er^3+^ ions in fluoroindate glasses co-doped with Pr^3+^/Er^3+^. The decay curves were measured under monitoring emission wavelength 980 nm and 1530 nm, respectively. In both cases, luminescence decay curves measured for Pr^3+^/Er^3+^ co-doped glass samples are shortened with increasing Pr^3+^ concentration in comparison to glasses singly doped with Er^3+^. Based on decay curves, luminescence lifetimes for the ^4^I_13/2_ and ^4^I_11/2_ excited levels of Er^3+^ were determined. Based on measured lifetimes for glass samples with the absence and presence of Pr^3+^, the energy transfer efficiencies for the ^4^I_13/2_ and ^4^I_11/2_ levels of Er^3+^ ions were also calculated. Luminescence lifetimes and energy transfer efficiencies varying with Pr^3+^ concentration are schematically presented on Fig. 8.

The luminescence lifetime for the ^4^I_13/2_ level of Er^3+^ is decreased from 8.60 ms (0.1Er) to 3.40 ms (0.1Er–0.1Pr), 1.96 ms (0.1Er–0.3Pr) and 1.36 ms (0.1Er–0.5Pr) with increasing Pr^3+^ concentration. The reduction of luminescence lifetime for the ^4^I_11/2_ level of Er^3+^ ions is also observed. The measured lifetime is changed from 6.65 ms (0.1Er) to 5.91 ms (0.1Er–0.1Pr), 4.92 ms (0.1Er–0.3Pr) and 4.14 ms (0.1Er–0.5Pr) for glass samples with the presence of Pr^3+^. Trends in luminescence lifetimes ^4^I_11/2_ and ^4^I_13/2_ (Er^3+^) varying with Pr^3+^ concentration are similar, but changes in lifetimes and their corresponding values for glasses singly doped with Er^3+^ and co-doped with Pr^3+^/Er^3+^ are significant from the spectroscopic point of view. It is worthy to notice that luminescence lifetimes obtained for Pr^3+^/Er^3+^ co-doped glasses are generally lower for the ^4^I_13/2_ level than ^4^I_11/2_ level, in contrast to measured lifetime for Er^3+^ singly doped glass which is higher for the ^4^I_13/2_ level (8.60 ms) than ^4^I_11/2_ level (6.65 ms). This is also the experimental proof that the intensity of near-infrared emission band at 980 nm due to the ^4^I_11/2_ → ^4^I_15/2_ transition increase, whereas the intensity luminescence band at 1535 nm related to the ^4^I_13/2_ → ^4^I_15/2_ transition of Er^3+^ is reduced with increasing Pr^3+^ concentration in fluoroindate glasses co-doped with Pr^3+^/Er^3+^. It was confirmed by luminescence decay curve measurements for similar fluoride ZBLAN glasses co-doped with Pr^3+^/Er^3+^ ions^41^, where the influence of the Pr^3+^ codopant on the ^4^I_13/2_ and ^4^I_11/2_ emission lifetimes has been also studied. For ZBLAN glasses, a significant reduction in the ^4^I_13/2_ lifetime was observed following the addition of a small amount of Pr^3+^, while the ^4^I_11/2_ lifetime of Er^3+^ was affected to a lesser degree. Thus, the degree of energy transfer process from Er^3+^ to Pr^3+^ ions is found to be much higher for the ^4^I_13/2_ level (ET2) than for the ^4^I_11/2_ level (Fig. 5). It was also confirmed by calculation of the energy transfer efficiencies in fluoroindate glasses co-doped with Pr^3+^/Er^3+^, where the values of η_ET_ are higher for the ^4^I_13/2_ level than for the ^4^I_11/2_ level. The energy transfer efficiency for the ^4^I_13/2_ level of Er^3+^ is increased from 60.5% (0.1Er-0.1Pr) to 77.2% (0.1Er-0.3Pr) and 84.2% (0.1Er-0.5Pr), whereas the value of η_ET_ for the ^4^I_11/2_ level of Er^3+^ is changed from 13.3% (0.1Er-0.1Pr) to 27.9% (0.1Er-0.3Pr) and 39.3% (0.1Er-0.5Pr) with increasing Pr^3+^ concentration.

**Figure 9:**
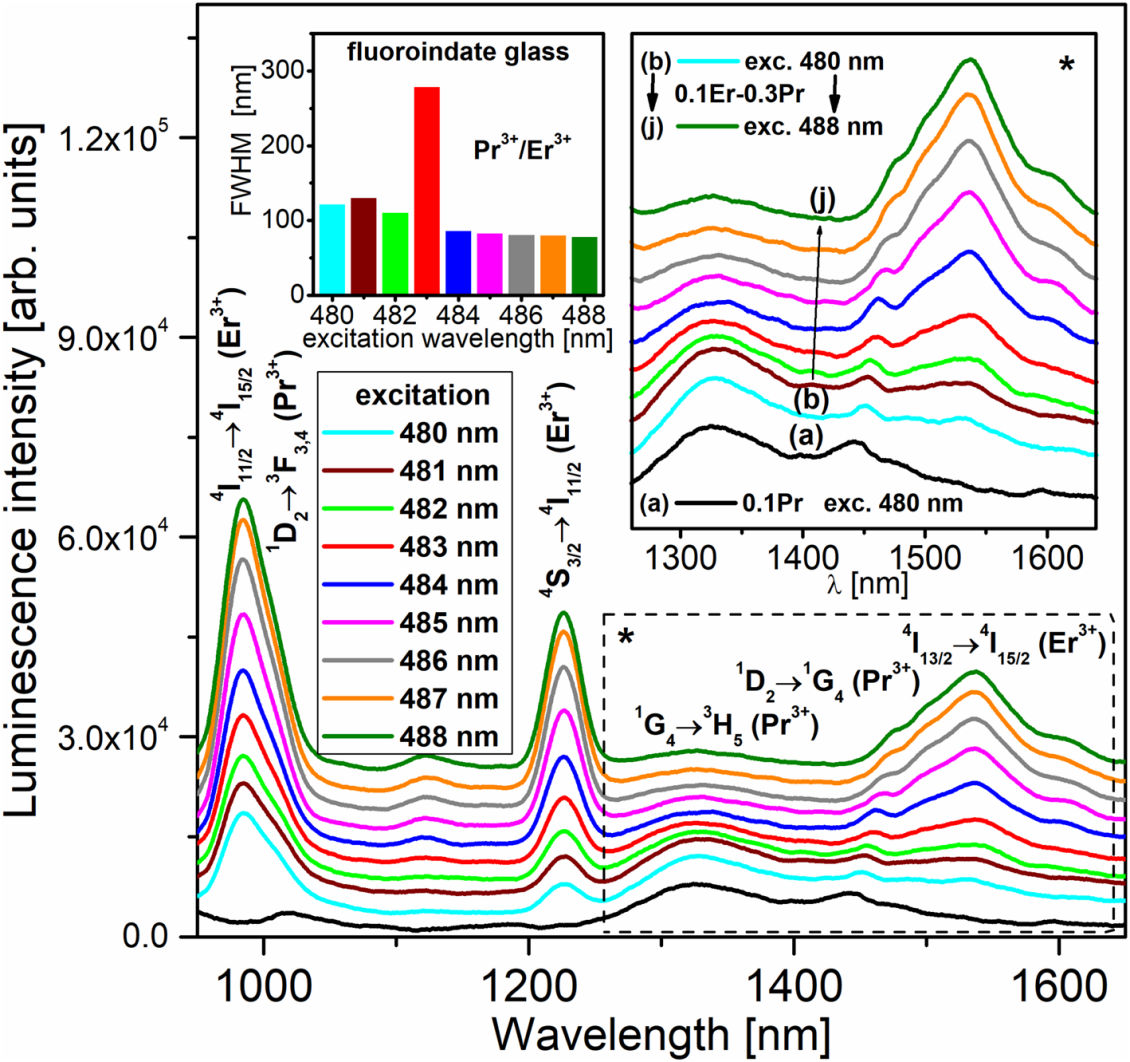
Near-infrared luminescence spectra of fluoroindate glasses co-doped with Pr^3+^/Er^3+^ under selective blue excitation. The inset shows values of FWHM depending on the excitation wavelength.

Figure 9 present near-infrared luminescence spectra of fluoroindate glasses co-doped with Pr^3+^/Er^3+^ under selective excitation wavelengths in the spectral range from 480 to 488 nm. This experiment motivated us to demonstrate unambiguously how the excitation wavelengths playing the crucial role in Pr^3+^/Er^3+^ co-doped system influence on broadband near-infrared luminescence covering a spectral range from 1250 to 1650 nm. The inset shows schematically values of emission linewidth (FWHM) depending on the selective excitation wavelengths.

The mechanisms for Pr^3+^/Er^3+^ co-doped glass system upon selective excitation wavelength at 488 nm (^4^F_7/2_ Er^3+^) involving several relaxation routes and two energy transfer processes ET1 (from the ^4^I_11/2_ level) and ET2 (from the ^4^I_13/2_ level) have been already discussed here.

**Figure 10:**
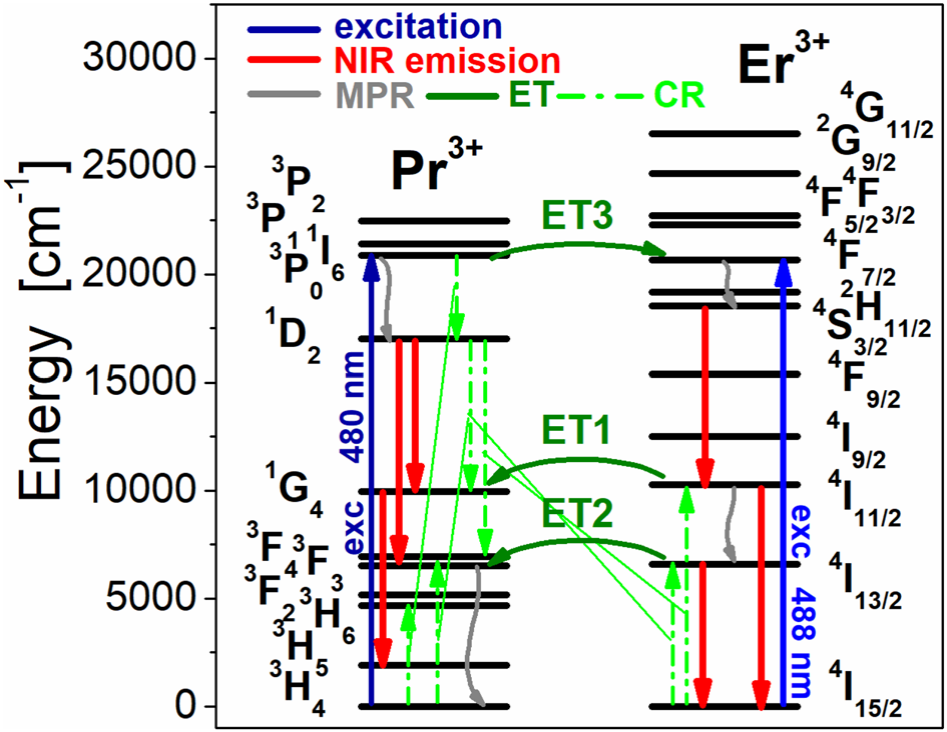
Energy level scheme and all transitions for fluoroindate glasses co-doped with Pr^3+^/Er^3+^ excited at blue spectral region.

Upon selective pumping at 480 nm, the ^3^P_0_ level of Pr^3+^ is well populated by the ground state absorption process (GSA) and then the excitation energy transfers by means of two ways. Part of the excitation energy is transferred from the ^3^P_0_ level of Pr^3+^ to the ^4^F_7/2_ level of Er^3+^ ions by the energy transfer process ET3. The second way to depopulate the ^3^P_0_ level is very fast non-radiative relaxation to the next lower-lying ^1^D_2_ level of Pr^3+^ ions through multiphonon relaxation (MPR) and cross-relaxation process (CR): ^3^P_0_, ^3^H_4_ → ^1^D_2_, ^3^H_6_. In the next step, several near-infrared emission bands originating to radiative transitions from the ^1^D_2_ and ^1^G_4_ levels to the lower-lying levels of Pr^3+^ are quite well observed and these aspects have been examined by us in this work. In particular, three near-infrared emission bands of Pr^3+^ and Er^3+^ located at spectral range denoted as (*) are the most interesting and important from the optical point of view. They are due to the ^1^G_4_ → ^3^H_5_ (Pr^3+^), ^1^D_2_ → ^1^G_4_ (Pr^3+^) and ^4^I_13/2_ → ^4^I_15/2_ (Er^3+^) transitions of rare earths in fluoroindate glass. Their relative intensities are changed drastically when the excitation wavelength varies from 480 to 488 nm. It is evidently to see that the luminescence band intensities of Pr^3+^ ions are reduced drastically, while the intensity of band due to the ^4^I_13/2_ → ^4^I_15/2_ transition of Er^3+^ ions is enhanced when the excitation wavelength is selectively changed from 480 to 488 nm. The inset of Fig. 9 shows values of FWHM depending on the excitation wavelength. When sample co-doped with Pr^3+^/Er^3+^ is excited at 480, 481 or 482 nm, the ^1^G_4_ → ^3^H_5_ transition of Pr^3+^ ions is dominant transition and emission linewidth seems to be 120–130 nm, which is similar to the value obtained for Pr^3+^ singly doped glass (FWHM = 130 nm). When glass sample co-doped with Pr^3+^/Er^3+^ is excited at spectral range 484/488 nm, the ^4^I_13/2_ → ^4^I_15/2_ transition of Er^3+^ ions is dominant transition and values of FWHM are close to about 75–85 nm, similar to the Er^3+^ singly doped glass (72 nm). Super-broadband near-infrared luminescence with corresponding value of FWHM = 278 nm is successfully observed, when Pr^3+^/Er^3+^ co-doped sample was excited selectively at 483 nm. The luminescence linewidth for Pr^3+^/Er^3+^ co-doped fluoroindate glass (278 nm) is similar to the values of FWHM equal to 236 nm and 296 nm, which were obtained for tellurite glasses doubly doped with Pr^3+^/Er^3+^ ions^42^ and triply doped with Pr^3+^/Er^3+^/Nd^3+^ ions^43^. Our experimental investigations confirm that the selective excitation wavelength 483 nm is an optimal pump source to obtain super-broadband luminescence in fluoroindate glass co-doped with Pr^3+^/Er^3+^. The previous results suggest that the Pr^3+^/Er^3+^ co-doped phosphate glasses with a 483 nm pump source exciting simultaneously both ^3^P_0_ (Pr^3+^) and ^4^F_7/2_ (Er^3+^) levels are promising amorphous materials for broadband optical amplifiers^44^. The energy level diagram and all transitions for fluoroindate glass co-doped with Pr^3+^/Er^3+^ excited selectively at blue spectral region are schematized on Fig. 10.

Based on previous scientific reports we confirm that our glass with Pr^3+^/Er^3+^ is a potential material for broadband fiber amplifiers^45^ and its near-IR emission property depends critically on the excitation wavelengths^46^. Similar to oxyfluoroaluminate and fluorozirconate systems^47^, fluoroindate glasses doped with rare earths have potential applications as laser materials.

**Figure 11:**
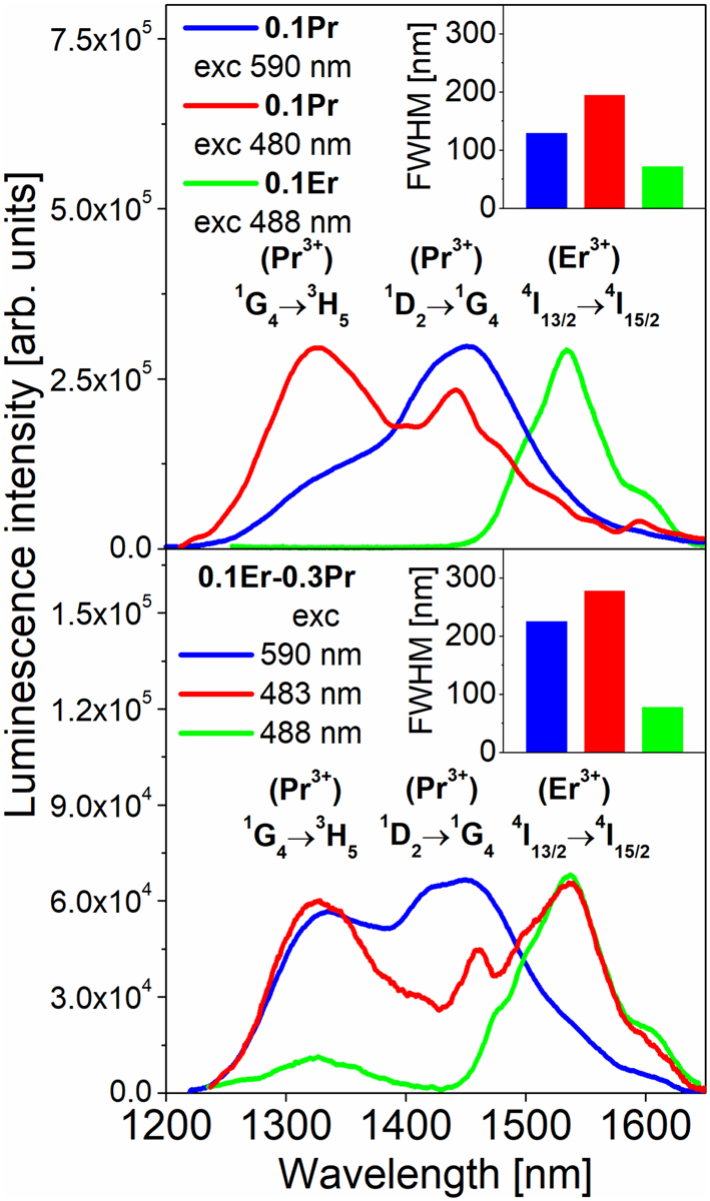
Normalized near-infrared emission spectra corresponding to the ^1^G_4_ → ^3^H_5_ (Pr^3+^), ^1^D_2_ → ^1^G_4_ (Pr^3+^) and ^4^I_13/2_ → ^4^I_15/2_ (Er^3+^) transitions of rare earth ions in fluoroindate glasses.

To summarize, near-infrared luminescence spectra of fluoroindate glasses co-doped with Pr^3+^/Er^3+^ at 1200–1650 nm range have been examined under different excitation wavelengths and the results are compared to glass samples singly doped with Pr^3+^ and Er^3+^ (Fig. 11).

Pr^3+^-singly doped fluoroindate glass shows near-infrared emission centered at 1335 nm and 1450 nm corresponding to the ^1^G_4_ → ^3^H_5_ and ^1^D_2_ → ^1^G_4_ transitions and their relative emission band intensities depend critically on the excitation wavelengths (480 and 590 nm). Er^3+^-singly doped fluoroindate glass under 488 nm excitation shows near-infrared emission near 1535 nm associated to the main ^4^I_13/2_ → ^4^I_15/2_ laser transition. When fluoroindate glass co-doped with Pr^3+^/Er^3+^ is excited at 590 nm, the intensity of emission band at 1335 nm due to the ^1^G_4_ → ^3^H_5_ transition is increased in comparison to the ^1^D_2_ → ^1^G_4_ transition at 1450 nm, and the spectral linewidth is larger for Pr^3+^/Er^3+^ co-doped glass sample (FWHM = 225 nm) than Pr^3+^ singly doped glass (FWHM = 130 nm).

Superbroadband near-infrared luminescence in Pr^3+^/Er^3+^ co-doped fluoroindate glass under selective excitation wavelength (483 nm) is successfully observed. Broadband emission with its linewidth equal to FWHM = 278 nm covering a wavelength range from 1200 to 1650 nm corresponds to the overlapped ^1^G_4_ → ^3^H_5_ (Pr^3+^), ^1^D_2_ → ^1^G_4_ (Pr^3+^) and ^4^I_13/2_ → ^4^I_15/2_ (Er^3+^) transitions of rare earth ions. However, we cannot unambiguously exclude the presence of ^3^F_3_,_4_ → ^3^H_4_ (Pr^3+^) transition at about 1600 nm, which was quite well observed in selenide glasses doped with Pr^3+^ and co-doped with Pr^3+^/Er^3+^ ions^48^. Further luminescent studies for Pr^3+^/Er^3+^ co-doped fluoroindate glasses excited at 488 nm suggest that they are promising candidates as dual-wavelength fiber-optic amplifiers for 1335 nm and 1535 nm windows similar to the previous excellent results published for Ge-As-Ga-S glasses co-doped with Pr^3+^/Er^3+^ ions^49^. Moreover reported emission properties of Pr^3+^/Er^3+^ and Er^3+^^50^ doped glasses inclined to use them in optical fiber construction as a fluoroindate fibers started to be valuable for SC generation^51^ and lasers^52–54^ beyond 3 μm. Future research will be devoted for fiber development and its length optimization as we believe to obtain superbroadband emission.

## Conclusions

Fluoroindate glasses co-doped with Pr^3+^/Er^3+^ have been investigated for near-infrared luminescence applications. Near-infrared luminescence properties have been examined under selective excitation wavelengths. The radiative and nonradiative relaxation channels involving several processes like multiphonon relaxation (MPR), cross-relaxation (CR), energy transfer up-conversion (ETU) and their mechanisms are proposed in Pr^3+^/Er^3+^ co-doped glass samples under direct excitation of Pr^3+^ and/or Er^3+^ ions and the energy transfer processes (ET) from Pr^3+^ to Er^3+^ and from Er^3+^ to Pr^3+^ were identified. In particular, near-infrared luminescence covering a wavelength range from 1200 to 1650 nm is really important for broadband optical amplifiers and these aspects for fluoroindate glasses have been analyzed in details. Broadband near-infrared emission (FWHM = 278 nm) corresponding to the ^1^G_4_ → ^3^H_5_ (Pr^3+^), ^1^D_2_ → ^1^G_4_ (Pr^3+^) and ^4^I_13/2_ → ^4^I_15/2_ (Er^3+^) transitions in fluoroindate glass co-doped with Pr^3+^/Er^3+^ is successfully observed under direct 483 nm excitation. Based on luminescence decay measurements, the measured lifetimes for the excited levels of rare earth ions and the energy transfer efficiencies were determined. Near-infrared luminescence spectra and their decays for glass samples co-doped with Pr^3+^/Er^3+^ are compared to the experimental results obtained for fluoroindate glasses singly doped with rare earth ions and theoretical calculations using the Judd–Ofelt framework.

## Methods

### Synthesis

Fluoroindate glasses singly and doubly doped with rare earth ions (Ln^3+^) with the following molar composition: 37.9InF_3_–20ZnF_2_–20SrF_2_–16BaF_2_–4GaF_3_–2LaF_3_–0.1LnF_3_, where Ln = Pr or Er (referred as 0.1Pr and 0.1Er), and (38–x–y)InF_3_–20ZnF_2_–20SrF_2_–16BaF_2_–4GaF_3_–2LaF_3_–xErF_3_–yPrF_3_, where x = 0.1; y = 0.1, 0.3, 0.5 (referred as 0.1Er–0.1Pr, 0.1Er–0.3Pr and 0.1Er–0.5Pr), were prepared by melting (covered platinum crucible) and quenching method in glove box in a nitrogen atmosphere (O_2_, H_2_O < 0.5 ppm). Ammonium bifluoride (NH_4_HF_2_) was added as afluorinating agentto the batch before melting. For the studied glass system, the concentration of ErF_3_ is relatively low (0.1 mol%). Thus, the energy transfer processes between Er^3+^ ions are negligibly small. With increasing ErF_3_ concentration the thermal stability reduces and strong luminescence quenching is observed due to nonradiative processes corresponding to the Er^3+^–Er^3+^ interaction increase. Also, previous investigations for fluoroindate glasses clearly indicate that the higher activator (Er^3+^) concentrations lead to partial crystallization of fluoroindate glasses^50^. The glass batches were firstly fluorinated at 270 °C for 2 h and then melted at 900 °C for 1 h. Finally, the glass was cast into a stainless steel plate and then annealed at 290 °C for 2 h slowly cooled to room temperature to minimize internal stress during the quenching process. Transparent glassy plates of 10 × 10 mm dimension were obtained. Each glass sample of 1 mm in thickness was polished for optical measurements.

### Measurement and characterization

Similar to our previous works for fluoroindate glasses^17,18^, the appropriate laser equipment was used for measurements of luminescence spectra and decay curves. The laser system consists of PTI QuantaMaster QM40 spectrofluorimeter, optical parametric oscillator (OPO), Nd:YAG laser (Opotek Opolette 355 LD), double 200 mm monochromators and multimode UVVIS PMT (R928) and Hamamatsu H10330B-75 detectors, and PTI ASOC-10 [USB-2500] oscilloscope. The Varian Cary 5000 UV–VIS–NIR spectrophotometer was used for the absorption spectra measurements and the Metricon 2010 prism coupler for the refractive index at a wavelength of 632.8 nm. Resolution for spectral measurements was 0.1 nm, whereas an accuracy for decay curves was ± 0.5 μs.

## Data Availability

All data regarding the work presented here is available upon reasonable request to the corresponding author.
